# Rapid Detection of Glycogen Synthase Kinase-3 Activity in Mouse Sperm Using Fluorescent Gel Shift Electrophoresis

**DOI:** 10.3390/s16040551

**Published:** 2016-04-16

**Authors:** Hoseok Choi, Bomi Choi, Ju Tae Seo, Kyung Jin Lee, Myung Chan Gye, Young-Pil Kim

**Affiliations:** 1 Department of Life Science, Hanyang University, Seoul 04763, Korea; chlghgh@nate.com (H.C.); charitybomi@gmail.com (B.C.); 2 Research Institute for Natural Sciences, Hanyang University, Seoul 04763, Korea; 3 Department of Urology, College of Medicine, Dankook University, Cheil General Hospital, Seoul 04619, Korea; jtandro@cgh.co.kr; 4 Department of Convergence Medicine, Asan Institute for Life Sciences, University of Ulsan College of Medicine, Asan Medical Center, Seoul 05505, Korea; kjlee@amc.seoul.kr; 5 Institute of Nano Science and Technology, Hanyang University, Seoul 04763, Korea; 6 Research Institute for Convergence of Basic Sciences, Hanyang University, Seoul 04763, Korea

**Keywords:** gel shift assay, GSK3, testis, sperm, isoelectric point, phosphorylation

## Abstract

Assaying the glycogen synthase kinase-3 (GSK3) activity in sperm is of great importance because it is closely implicated in sperm motility and male infertility. While a number of studies on GSK3 activity have relied on labor-intensive immunoblotting to identify phosphorylated GSK3, here we report the simple and rapid detection of GSK3 activity in mouse sperm using conventional agarose gel electrophoresis and a fluorescent peptide substrate. When a dye-tethered and prephosphorylated (primed) peptide substrate for GSK3 was employed, a distinct mobility shift in the fluorescent bands on the agarose was observed by GSK3-induced phosphorylation of the primed peptides. The GSK3 activity in mouse testes and sperm were quantifiable by gel shift assay with low sample consumption and were significantly correlated with the expression levels of GSK3 and p-GSK3. We suggest that our assay can be used for reliable and rapid detection of GSK3 activity in cells and tissue extracts.

## 1. Introduction

Glycogen synthase kinase-3 (GSK3) is a constitutively active serine/threonine kinase found in all eukaryotes with a high degree of homology [1]. Changes in the GSK3 activity in response to diverse stimuli is implicated in numerous cellular processes, including glycogen metabolism, gene transcription, vesicular transport, cell division, and apoptosis [2,3,4]. Recently, GSK3 (especially GSK3α) has been shown to play a key role in sperm motility and the knockout of GSK3 leads to a significant reduction in mouse sperm motility, which is closely linked to male infertility [5,6,7]. Compared to immobile spermatozoa, there is a high degree of inhibitory phosphorylation of GSK3 in motile epididymal spermatozoa, resulting in lower GSK3 activity [8]. With increasing attention to the role of male infertility in the field of human reproduction, a reliable analysis of GSK activity can serve as an improved diagnostic approach for male infertility, which traditionally has depended on time-consuming observations of sperm morphology and behavior. However, despite recent advances in bioassay technology, there have not been any attempts to rapidly detect GSK3 activity in sperm. GSK3 activity is governed either by the inhibitory phosphorylation of its serine residue (Ser21 in GSK3α and Ser9 in GSK3β) or by induced changes in substrate accessibility or recognition through phosphorylation of its tyrosine residue (Tyr279 in GSK3α and Tyr216 in GSK3β) [9,10], past approaches to detecting GSK-3 activity in cells or tissues have relied on immunoblotting with GSK3 phospho-specific antibodies, which is time-consuming and not always reflective of endogenous GSK3 activity [11]. To circumvent this problem, we previously reported that the use of GSK3-specific peptide substrate, the isoelectric focusing (IEF) technique, and microfluidics enabled the easy detection of the GSK3 activity [12]. In this method, the prephosphorylated (primed) peptide labeled with a fluorescent dye was sequentially phosphorylated in response to GSK3, leading to the separation of peptide substrates in the fluorescent IEF region, due to the increased negative charge of the phosphate groups of the primed peptide. In comparison to immunoblotting, this IEF-based method can provide us with a better understanding of phosphorylation degree and the mean difference between the unphosphorylated and phosphorylated substrates. We belive that there would be great interest in this assay process if we could improve the detection detection system.

Here we report the simple and rapid detection of GSK3 activity in mouse sperm using a fluorescent gel mobility shift assay on agarose. While electrophoretic mobility shift assays (EMSAs) have long been used to probe DNA-protein [13,14], RNA-protein [15], and peptide-protein interactions [16], there have only been a few studies on enzyme-substrate interactions, especially those using a non-radioactive fluorescent method on agarose. Moreover, unlike a microfluidic (or gel-type) IEF device, simple gel electrophoresis, which consist of a conventional gel electrophoresis apparatus, an agarose, and an ultraviolet (UV) illuminator, does not require a complicated fabrication process to generate the microfluidic channels or focused conditions to generate a pH gradient. With appropriate substrates to induce a dramatic mobility change in response to enzyme activity, it is possible to design a powerful protein kinase assay from this gel mobility shift method. Many efforts to improve protein kinase assays have used nanoparticle-combined methods through colorimetric or fluorescent detection, but most intrinsically suffer from a high degree of non-specific signals. For example, we previously reported that different types of nanosensors showed great simplicity and/or detection sensitivity to protein kinase or phosphatase activity [17,18], but were stringently limited to biological media due to the presence of strong non-specific signals. These methods thus require inconvenient pre-treatment steps such as immunoprecipitation or affinity pull-down. To address this shortcoming, we believe that fluorescent gel shift assay using dye-tethered peptides can be an alternative and simpler approach to assaying GSK3 activity as compared to immunoblotting, IEF-based electrophoresis, and other protein kinase assays. Considering that isoelectric point (p*I*)-based separation and visualization of fluorescent peptides can quantify GSK3 activity by simple imaging analysis, we examined the activity of endogenous GSK3 in mouse testis and epididymal sperm extracts, which was compared with the results of immunoblotting using GSK3 and GSK3 phosphorylation antibodies.

## 2. Materials and Methods

### 2.1. Materials

Recombinant glycogen synthase kinase-3 (GSK3) was purchased from New England Biolabs (Hitchin, UK). Lithium chloride (99%, LiCl) and adenosine 5′-triphosphate (ATP) disodium salt hydrate (99%, 5′-ATP-Na_2_) were purchased from Sigma-Aldrich (Yongin, Korea). Tetramethyl-6-carboxyrhodamine (TAMRA)-labeled peptides (T-Pep, TAMRA-KEEPPSPPQSPR; T-Pep(p), TAMRA-KEEPPSPPQpSPR; T-Pep(pp), TAMRA-KEEPPpSPPQpSPR, where the underlined letters indicate the phosphorylation site) were synthesized from Peptron, Inc. (Daejeon, Korea). Antibodies to GSK3α/β (#5676) and phospho GSK3α/β (#9327) were from Cell Signaling Technology (Beverly, MA, USA). Horseradish peroxidase-conjugated goat anti-rabbit IgG (ab6721) and Alexa488-conjugated donkey anti-rabbit IgG (ab150061), and rabbit normal IgG (ab172730) were from Abcam (Cambridge, UK). The enhanced chemiluminescence (ECL)-Plus kit was from Amersham-Pharmacia (Buckinghamshire, UK). Protease inhibitor cocktail Xpert was from GenDEPOT (Barker, TX, USA). All other chemicals were of analytical grade and were used as received.

### 2.2. Fluorescent Gel Shift Assay on Agarose

Unless otherwise stated, all gel shift assays were performed on 1% agarose at 50 V for 60 min. The agarose gel was cast and run using 1 × Tris-Borate (TB) buffer without ethylenediaminetetraacetic acid (EDTA). For the kinase activity assay, T-Pep(p) (1 μL at 100 μM), GSK3 (1 μL at 25 U), ATP (0.4 μL at 10 mM), and 10 × kinase reaction buffer (2 μL) were mixed at a final volume of 20 μL in standard reaction buffer (20 mM Tris-HCl, pH 7.4). The reactants were typically incubated in a tube for 120 min at 30 °C to induce the enzyme reaction, and then were loaded into the agarose gel. As a control, a solution of single peptide (1 μL at 100 μM) or a mixed solution of the three peptides (T-Pep, T-Pep(p), and T-Pep(pp)) in a 1:1:1 molar ratio was used in the gel shift assay in a final volume of 20 μL in 20 mM Tris-HCl (pH 7.4). For the kinase inhibition assay, a stock solution of LiCl was serially diluted to different concentrations in the reaction buffer, and an equal volume (2 μL) of each inhibitor concentration was then mixed with a solution containing T-Pep(p), GSK3, and ATP dissolved in the reaction buffer. For the GSK3 activity assay in the mouse tissue extract, an appropriate volume of crude sample was diluted in a RIPA buffer (Cell Signaling Technology) to adjust to the same total protein concentration in the lysates. A 5 μL aliquot of lysate was mixed with 1 μL of 100 μM T-Pep(p) and 0.4 μL of 10 mM ATP at a final volume of 20 μL in the reaction buffer (20 mM Tris-HCl, pH 7.4). The reactants were further incubated for 120 min at 30 °C and then underwent gel electrophoresis. The fluorescent image of the agarose was taken on a UV transilluminator equipped with a digital camera (UVP Inc., Upland, CA, USA). The original image underwent a pseudo-color imaging process.

### 2.3. Determination of GSK3 Activity

The relative GSK3 activity was calculated using the following equation:

(1) $$\mathrm{GSK}3\mathrm{Activity}\;(\%)=\frac{I_{\mathrm{Band}(p)}}{I_{\mathrm{Band}}+I_{\mathrm{Band}(p)}}\times 100$$

where *I*_Band_ and *I*_Band(p)_ are the fluorescent intensities of the control band (before the GSK3 reaction) and the phosphorylated band (after the GSK3 reaction) on agarose, respectively. The fluorescent intensities were analyzed using ImageJ software (USA National Institutes of Health, Bethesda, MD, USA).

### 2.4. Sperm Collection

The epididymides were dissected from adult male mice under CO_2_ asphyxiation. Cauda epididymides were cut and squeezed to release the sperm into Tyrode’s complete medium (TCM) containing 0.3% bovine serum albumin [19]. After suspending and incubation for 10 min at room temperature the motile sperm fraction was aspirated and washed by centrifugation at 500× *g* for 5 min. At the end of the incubation sperm was subjected to a GSK3 activity assay and western blotting.

### 2.5. Localization of GSK3 in the Testis and Sperm

Adult mouse testis fixed in Bouin’s solution were embedded in paraffin and 5 μm-thick sections were subjected to immunohistochemical localization of GSK3 using the detection antibody GSK3α/β diluted 1:1000 in blocking solution (3% donkey serum in phosphate-buffered saline (PBS)). After several washes HRP-conjugated goat anti-rabbit IgG diluted 1:100 in blocking solution was applied for 1 h. Signal was developed with 3,3′-diaminobenzidine (DAB). For immunocytochemical localization of GSK3 in mature sperm, cauda epididymal sperm that was dry-smeared on a poly-L-lysine coated slide was fixed in acetone for 10 s and subjected to labeling with anti-GSK3 antibody diluted 1:100 in blocking solution. As a negative control, rabbit normal IgG was used. Signal was developed with Alexa488-conjugated donkey anti-rabbit IgG diluted 1:200 in blocking solution. The samples were wet mounted with Prolong Gold containing 4′,6-diamidino-2-phenylindole (DAPI) for nuclear staining and the images were captured using a fluorescence microscope system equipped with cooled CCD (DP71, Olympus, Tokyo, Japan).

### 2.6. Western Blotting

Tissue were sonicated for 5 s at 4 °C in PBS containing 1% Triton-X-100 and 1% (V/V) protease inhibitor cocktail. Cell lysates were resolved in duplicate by SDS-PAGE and transferred to nitrocellulose membranes. Western blotting was performed using GSK3α/β and phospho GSK3α/β antibodies (1:10,000 in 5% skim milk) and horseradish peroxidase-conjugated goat anti-rabbit IgG (1:5000 in 5% skim milk). Chemiluminescence detection was performed with the ECL detection kit according to the manufacturer’s instructions.

## 3. Results and Discussion

### 3.1. GSK3 Activity Assay on Agarose

**Figure 1:**
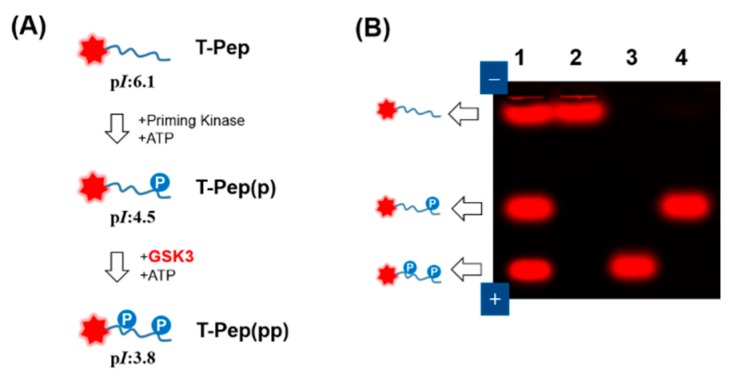
(**A**) Schematic of phosphorylation-induced change in the p*I* of peptide substrate for GSK3 activity assay. T-Pep, T-Pep(p) and T-Pep(pp) represent TAMRA-KEEPPSPPQSPR, TAMRA-KEEPPSPPQpSPR, and TAMRA-KEEPPpSPPQpSPR, respectively; (**B**) Fluorescent gel image from electrophoretic mobility shift assay of GSK3 activity in a reaction buffer. Lane 1, a control mixture of T-Pep, T-Pep(p), and T-Pep(pp) at a 1:1:1 molar ratio; Lane 2, a mixture of T-Pep and GSK3 with ATP; Lane 3, a mixture of T-Pep(p) and GSK3 with ATP; Lane 4, a mixture of T-Pep(p) and GSK3 without ATP. Each sample was separated on a 1% agarose gel at 50 V for 60 min in 1 × TB. The final concentrations of fluorescent peptide and recombinant active GSK3 were 5 μM and 1.25 U, respectively. The gel was photographed on a UV transilluminator.

To assay GSK3 activity, we chose a peptide substrate (KEEPPSPPQSPR) derived from heat shock transcription factor 1 (HSF1), which is known to be consecutively controlled by two protein kinases: p42 mitogen-activated protein kinase (MAPK) and GSK3 [20,21]. As depicted in Figure 1A, the priming kinase (e.g., MAPK) can phosphorylate the last Ser residue of the peptide in the presence of ATP, subsequently causing the GSK3 to induce the phosphorylation cascade at the first Ser residue. While Ser/Thr-X-X-X-Ser/Thr-Pro was reported to be a consensus sequence of the substrate for GSK3 [22], it is important to note that the last Ser-Pro/Thr-Pro generally induces priming phosphorylation by the priming kinase, which is followed by GSK3-induced phosphorylation because GSK3 has an unusual preference for priming phosphorylation [23]. While DNA electrophoresis is dependent on sample size, charge, and shape, the proposed peptide electrophoresis shows only the charge difference in peptide status, causing the phosphorylated form to shift down on the gel toward the positive electrode. To determine whether the prephosphorylated (primed) peptide substrate is specific for GSK3 activity and whether the agarose gel mobility shift assay can detect this phosphorylation event, the mobility of the peptide substrate was examined on agarose before and after the substrate-enzyme reaction. For easy and sensitive visualization, three types of peptide substrates were synthesized by tethering 5(6)-carboxytetramethylrhodamine (TAMRA) at their N-termini: TAMRA-KEEPPSPPQSPR (termed T-Pep), TAMRA-KEEPPSPPQpSPR (termed T-Pep(p)), and TAMRA-KEEPPpSPPQpSPR (termed T-Pep(pp)). Taking into consideration the expected p*I* values of the sequentially phosphorylated peptide (6.1 for T-Pep, 4.5 for T-Pep(p), and 3.8 for T-Pep(pp)), which were calculated from the mean p*K*a values for the phosphorylated amino acids, one would expect this peptide substrate to be well-separated at a neutral pH. As shown in Figure 1B, when the control mixture of three dye-tethered peptides was loaded onto 1% agarose in a 1:1:1 molar ratio, three strong fluorescent bands on agarose were clearly separated and visualized on a UV transilluminator (Lane 1), showing the peptide’s degree of phosphorylation. Significantly, no band shift was observed for non-primed peptide (T-Pep) (Lane 2) and ATP-deficient reactions (Lane 4), whereas a strong band shift was observed only when the T-Pep(p) was reacted with GSK3 in the presence of ATP (Lane 3). This result clearly shows that the primed peptide (T-Pep(p)) is suitable for assaying GSK3 activity using the fluorescent gel shift method.

**Figure 2:**
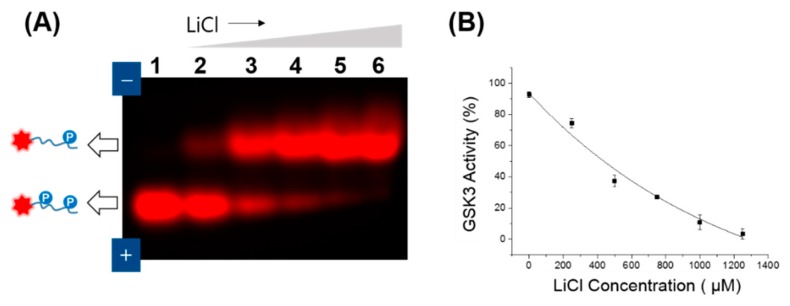
Fluorescent gel shift-based inhibition assay of GSK3 activity as a function of inhibitor (lithium chloride, LiCl) concentration: (**A**) Fluorescent gel image from the GSK3 inhibition assay using LiCl (0−1.25 mM) in the presence of T-Pep(p). T-Pep(p) was reacted with recombinant active GSK3 at varied concentrations of LiCl: 0 μM (Lane 1), 200 μM (Lane 2), 500 μM (Lane 3), 750 μM (Lane 4), 1 mM (Lane 5), and 1.25 mM (Lane 6); (**B**) Inhibitory graph of GSK3 activity as a function of LiCl concentration. GSK3 activity (%) was calculated and plotted from the relative fluorescence intensities in the two band regions which corresponded to T-Pep(p) and T-Pep(pp) on agarose, according to Equation (1). The final concentration of GSK3 was 1.25 U in 20 μL of reaction volume. The standard deviation was obtained from three independent experiments.

To investigate whether GSK3 activity is specifically regulated, GSK3 inhibitor assay was performed using fluorescent gel shift assay. Because lithium (Li^+^) is a competitive inhibitor of GSK3 with respect to magnesium (Mg^2+^) [24], lithium chloride (LiCl) was treated with GSK3 at various concentrations. As shown in Figure 2A, compared to the control (lane 1, without LiCl), the fluorescent intensity at the T-Pep(p) band notably increased as the LiCl concentration increased (from Lane 2 to Lane 6). Using Equation (1) from the Materials and Methods to calculate the relative fluorescence intensity of T-Pep(pp) over the summation of T-Pep(p) and T-Pep(pp) on the agarose band, Figure 2B plots the dose-dependent GSK activity. While the maximum attainable GSK3 activity was 92.6%, the half maximal inhibitory concentration (IC_50_) value of LiCl was determined to be 438 μM, which was much lower than the concentration in an ealier report (an IC_50_ of 2.5 mM) [24]. This result suggests that the electrophoretic gel shift assay can be used to quantify GSK activity with high accuracy.

### 3.2. Expression, Kinase Activity and Phosphorylation of GSK3 in Sperm

**Figure 3:**
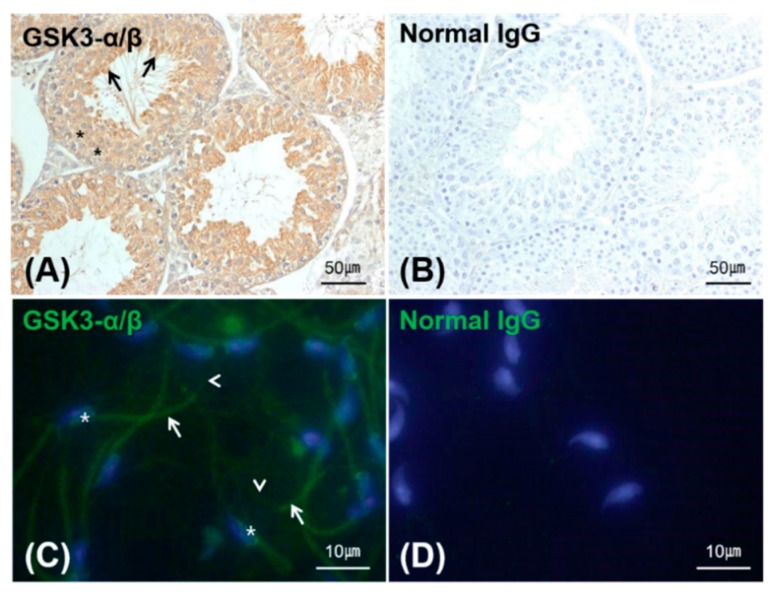
Representative images of of GSK3α/β immunohistochemistry staining in mouse testis (**A**,**B**) and epididymal sperm (**C**,**D**). Anti-GSK3α/β antibody was used for visualizing the localization of GSK3α/β (**A** (Brown), **C** (Green)), whereas normal IgG was used as a negative control (**B**,**D**). Brightfield (**A**,**B**) and fluorescence images (**C**,**D**) were obtained from simulated HRP-conjugated secondary antibody/DAB and Alexa488-conjuaged secondary antibody, respectively. In the testis, GSK3 was expressed in the Sertoli cells (asterisks) and developing germ cells including sperm (arrows); in epididymal sperm GSK3 (green) was expressed in the head (asterisks), midpiece (arrows), and principal piece of the tail (arrowheads). Nuclei were stained blue by DAPI.

To investigate the localization of GSK3 in mouse tissue, immunohistochemical staining of GSK3 expressed in the seminiferous tubule was performed, in which Sertoli cells and developing germ cells including sperm showed GSK3 immunoreactivity (Figure 3A). In mature sperm, strong GSK3 immunoreactivity was found in the post-acrosomal region of the head and midpiece (Figure 3C). The principal piece of the tail was weakly positive for GSK3 immunoreactivity. No significant immunoreactivity was observed using the negative control antibody (Figure 3B,D). This result is in accordance with previous reports in the literature that GSK3 is present in the head and tail regions of sperm [8].

**Figure 4:**
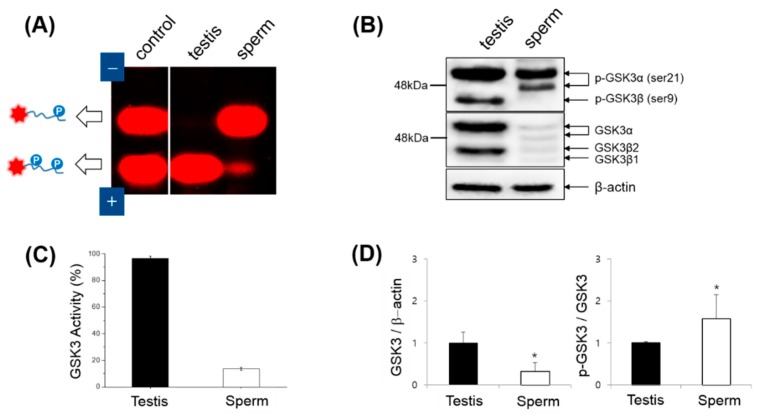
(**A**) Fluorescent gel shift assay of GSK3 activity in mouse testis and sperm extracts. T-Pep(p) was used to assay endogenous GSK3 activity after being mixed with testis or sperm extract at the same total protein concentration. (**B**) Western blots developed with anti-phosphoserine (Ser9/Ser21 in GSK3) antibody (Top) and anti-GSK3α/β antibody (Middle) in the testis and sperm extracts. The control anti-β-actin antibody (Bottom) was used as a control to ensure equal protein loading. The relative GSK activity (%) (**C**), GSK expression (GSK3/β-actin, left graph in (**D**)), and GSK inhibition (p-GSK3/GSK3, right graph in (**D**)) were analyzed from (**A**,**B**). The standard deviations in (**C**,**D**) were obtained from three independent experiments.

With this localization of GSK3 in mind, we examined both the activity and expression of GSK3 in mouse extracts using two *in vitro* assays (Figure 4). Upon the addition of soluble mouse extracts to the primed peptide substrate (T-Pep(p)) for GSK3, a significant difference in fluorescent gel mobility between the testis and sperm extracts was observed, indicating higher GSK3 activity level in the testis than in the sperm (Figure 4A). The calculated GSK activity was revealed to be 7-fold higher in the testis than in the sperm (Figure 4C), which may be attributed to the relatively high expression of GSK3 in the testis or the relatively high inhibitory phosphorylation of GSK3 in sperm. To investigate these hypotheses, we performed western blot analysis of GSK3 in mouse testes and epididymal sperm (Figure 4B). Since there are two mammalian GSK3 isoforms (GSK3α and GSK3β) that are encoded by distinct genes [25], multiple bands of MW ranging from 44 to 51 kDa were observed using anti-GSK3α/β antibody. It should be noted that the difference in size between the two isoforms is due to a glycine-rich extension found only in the amino terminus of GSK3α [11]. Two bands of MW 44–46 kDa corresponding to GSK3β were detected in the sperm. In mammals two splicing variants of GSK3β have been reported [26], suggesting that these bands may represent the GSK3β variants. In contrast, a 51-kDa band corresponding to GSK3α was detected together with a 48 kDa band in sperm. Currently, it is not clear whether the 48 kDa band is the cleaved product of GSK3α, but this result supports previous evidence that isoforms of both GSK3α and GSKβ are present in spermatozoa, with GSK3α being the predominant isoform [7]. On western blots, the total amount of GSK3 in the testis lysate was much greater than the amount in the sperm lysate, whereas the p-GSK3/GSK3 ratio of the sperm extract was much greater than that of the testis (Figure 4D). Taking into account the strong GSK3 immunoreactivity in somatic cells and developing germ cells in the testis, highly phosphorylated GSK3α might be a unique feature of mature sperm. In addition, GSK3β was highly phosphorylated in Ser9 in the testis but not in mature sperm. Despite low GSK3/actin ratio in mature sperm, the high level of phosphorylation of the Ser21 of GSK3α is a unique feature of mature sperm which might be responsible for the low GSK3 activity. Based on the crucial role of GSK3 in sperm maturation and fertility [27], the changes in the phosphorylation of Ser21 of GSK3α might be important for the role of sperm fertilization. This result indicates that the endogenous GSK3 activity in mouse sperm extracts can be easily quantified using fluorescent gel shift assay.

Despite the recent development of protein kinase assays [28,29], general chemical probes [30,31], or enzymatic signal generation [32], our approach using fluorescent peptides represents a significant innovation enabling the simple and easy assay of specific enzymatic activity. Particularly when compared to labor-intensive techniques such as immunoblotting and radioactive-based assays, this method allows for rapid assay (less than 2 h), low sample consumption (20 μL), and accurate quantification of protein kinase activity by ratiometric imaging analysis. Moreover, biological media can be applied to this assay pretreatment and in a way that minimizes the loss of cellular components. In contrast, immunoprecipitation or affinity-based pull-down processes generally result in a massive loss of endogenous target proteins in the crude sample. In this regard, our approach is expected to allow for the simple and rapid detection of GSK3 activity in sperm and holds great potential for further advances in assaying protein kinase activity.

## 4. Conclusions

We demonstrated the detection of GSK3 activity in mouse testis and epididymal sperm using a simple gel shift assay, based on the change in p*I* of a fluorescent peptide substrate. Without requiring complicated devices or a labor-intensive process, this method using simple agarose gel electrophoresis enabled us to assay the endogenous GSK3 activity in tissue extracts and quantify the inhibitory effect of GSK activity. Owing to its general usability, we anticipate that this assay will serve as an effective and efficient means for evaluating GSK3 activity in sperm, facilitating to the study of the physiological roles of GSK3.
